# Resistance training induces similar adaptations of upper and lower-body muscles between sexes

**DOI:** 10.1038/s41598-021-02867-y

**Published:** 2021-12-06

**Authors:** Filip Kojić, Danimir Mandić, Vladimir Ilić

**Affiliations:** 1 Teacher Education Faculty, University of Belgrade, Kraljice Natalije 43, Belgrade, 11000 Serbia; 2 Faculty of Sport and Physical Education, University of Belgrade, Belgrade, Serbia

**Keywords:** Physiology, Anatomy

## Abstract

The purpose of the study was to compare sex adaptations in hypertrophy, strength and contractile properties of upper and lower-body muscles induced by resistance training (RT). Eighteen RT untrained male (MG) and female (FG) students (aged 24.1 ± 1.7 years, height: 1.75 ± 0.08 m, weight: 70.4 ± 12.3 kg) undervent 7 weeks of biceps curl and squat training (2 days/week, 60–70% repetition maximum, 3–4 sets, 120 s rest intervals, reps until muscular failure). At baseline and final measurement, thickness and cross-section area, one-repetition maximum and tensiomyography parameters (contraction time − Tc and radial displacement − Dm) of elbow flexors (biceps brachii) and knee extensors (4 quadriceps muscles) were evaluated. Although MG tends to display greater absolute strength gains for upper- (p = 0.055) and lower-body (p = 0.098), for relative changes ANCOVA revealed no sex-specific differences for either of the tested variables. Significant hypertrophy was observed for all tested muscles, except for vastus intermedius in FG (p = 0.076). The Dm significantly decreased for biceps brachii (MG by 12%, p < 0.01 and FG by 13.1%, p < 0.01) and rectus femoris (MG by19.2%, p < 0.01 and FG by 12.3%, p < 0.05), while Tc values remain unchanged. These results indicate that initial morphological, functional and contractile alterations following RT are similar for males and females, and that there are no specific sex adaptations either for the upper- or lower-body muscles. The study was registered with ClinicalTrials.gov (NCT04845295).

## Introduction

It is well-documented that males and females differ on the anatomical and physiological level. For instance, there are known sex variations in limb length and pelvic angle, muscle size, and general body-composition, hormonal fluctuations, fatigability, and inflammatory response after exercise prescription^1–3^. Thus, there is a physiological rationale that resistance training (RT) could produce different muscular adaptations between the sexes.

Previous RT studies were mainly focused on hypertrophy and strength gains. Considering the sex differences in the baseline muscularity and force production^3^, it is not surprising that males often exceed females in an absolute increase of muscle mass and strength^4–6^. However, in regard to relative changes, the findings are equivocal. Many researchers have evaluated lower-body muscles (quadriceps femoris) and while some^2,5^ noted significant differences between older men and women, others^4,6–10^ reported similar relative gains in hypertrophy and strength between sexes, for both younger and older population. On the other hand, less attention has been paid to upper-body muscles. Based on studies^11–14^, it seems that females exhibit greater strength changes. Indeed, in a recent meta-analysis Roberts et al.^15^ concluded that RT induces similar muscular adaptations between young-adult untrained males and females for lower-body muscles. Yet, for relative upper-body strength increase, the effect size was moderately in favor for females. In that context, it is plausible that neural adaptations in females are more pronounced during the first weeks of RT for upper-body muscles^15,16^, resulting in higher relative strength gains. Although this potentially indicates that strength adaptations between sexes differ according to the specific body region, few studies included both upper- and lower-body muscles and analogously evaluated their absolute and relative changes in hypertrophy and strength. Furthermore, when it comes to lower-body muscles, evaluation of quadriceps muscles on individual level, except vastus lateralis, is lacking. This is important, since males and females show different kinematic and muscle activity patterns during lower-body movement^1,17–19^, which could results in non-homogeneous (intermuscular) hypertrophy of quadriceps femoris^20^. Namely, rectus femoris tends to display sustainably higher activity in females compared to males during one-legged squat^17–19^ and knee extension^1^ fatiguing exercise. Moreover, sex differences have been noted in intermuscular development of quadriceps muscles regarding similar sport participation, where regular rowing activity caused preferential growth of vastus medialis in females, and inversely vastus lateralis in males^21^. Thus, there is a need to consider all quadriceps parts (rectus femoris and vastii muscles) when aiming to clarify sex comparison following lower-body RT prescription.

Except hypertrophy and strength changes, evaluating passive mechanical properties may provide additional insight into adaptive processes of skeletal muscle^22^. This particularly relates to tensiomyography (TMG) and two parameters extracted by this method: contraction time (Tc) and radial displacement (Dm). The Dm, which represents muscle belly stiffness^22^, is sensitive to RT stimulus and it has been shown that RT leads to reduced Dm values (i.e. higher stiffness) both acutely^23^ and chronically^24^. Accordingly, chronic changes in Dm values have been associated with muscle morphological adaptations, indicating that Dm could be used to detect RT effectiveness for hypertrophy^24,25^. On the other hand, Tc has been related to fiber type proportions, where lower values of the Tc have been correlated with slow twitch muscle fibers^26^. Hence, in addition to hypertrophy and strength changes, TMG evaluation could give comprehensive information regarding sex specific adaptations following RT. Nevertheless, a recent review by Lohr et al.^22^ points out that only 13% of female subjects participated in the previous TMG research and there is a paucity of data on sex differences in the contractile properties of various muscle groups. To the best of the authors’ knowledge, only few studies have addressed this issue and all had a cross-sectional research design^27,28^. While their findings indicated sex dissimilarities in the Dm for some lower-body (biceps femoris and rectus femoris) muscles, there is a dearth in the literature regarding whether RT produces different chronic TMG adaptations between males and females.

In the current study we aimed to compare biceps curl and parallel squat RT effects between young men and women on: (i) muscle thickness (MT) and cross-section area (CSA) of biceps brachii and 4 quadriceps femoris muscles, (ii) one-repetition maximum (1RM) of elbow flexor and knee extensors, (iii) TMG Dm and Tc of biceps brachii, rectus femoris and vastus lateralis muscle. We hypothesized that: (i) squat RT would produce inhomogeneous hypertrophy of quadriceps muscles between males and females, (ii) relative gains in 1RM of elbow flexors would be greater in females compared to males, (iii) decrease in Dm values of quadriceps muscles would be different between the sexes.

## Methods

### Experimental approach to the problem

**Figure 1:**
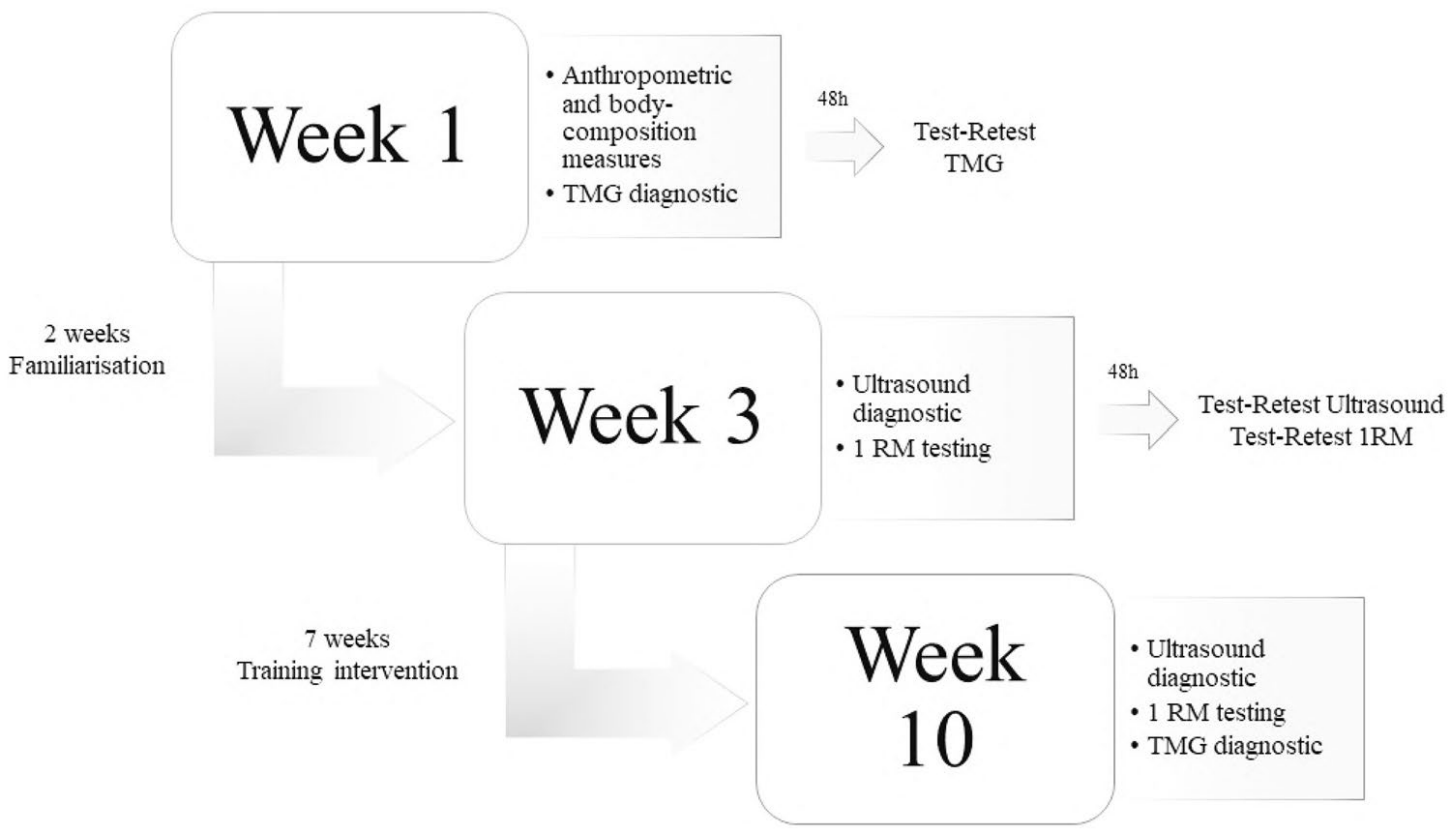
Schematic figure of study design. Graphical representation was designed using Adobe Photoshop software version 23.0.1 (https://www.adobe.com/products/photoshop.html).

The participants were assigned to two experimental groups, based on the sex (MG—male group and FG—female group). The training intervention was performed twice a week over a period of for 7 weeks. Before training intervention, a 2-week familiarization period was conducted to ensure that subjects have mastered the technique of selected exercises^29^. Elbow flexor and knee extensors muscle dimension and strength were assessed 2 days before and after the training intervention, while contractile properties were evaluated 5 days before and after the experiment^30^. Participants were blinded for test results. Flowchart of experimental procedure is presented in Fig. 1.

### Subjects

The sample included twenty-four moderately active university students (12 females and 12 males), who have not participated in RT activity in the previous 8 months. Thus, according to Santos Junior et al.^31^, the included participants could be classified as beginners in RT. Six participants were excluded (3 males and 3 females), due to loss of interest and personal issues. The final sample included 18 participants (9 men and 9 women) who successfully completed the experimental protocol. A priori sample size was justified utilizing G-Power software (University of Kiel, Kiel, Germany, version 3.1), using biceps brachi thickness as the outcome measure with a target effects size of 0.75 for untrained population^32^, alpha level of 0.05 and a power (1 − ß) of 0.8. The sample characteristics, including age, body composition and relative strength index (lifted load/body mass ratio), for each sex are presented in Table 1. Body height was taken using portable Martin’s anthropometer (Siber-Hegner, Switzerland), with 0.1 cm accuracy. Body composition variables were measured by In-Body 720 (Biospace Co., Seoul, Korea) using Direct Segmental Multi frequency–Bioelectrical Impedance Analysis (DSM–BIA method). Prior to testing, the subjects were instructed not to eat anything in the morning, to avoid any kind of exercise 24 h before body composition analyses and to meet their physiological needs before the measurement. Subjects were in the standing position for at least 5 min prior to measurement for redistribution of body fluids. During the measurement all subjects were in light sport clothing and had no metal accessories.

**Table 1:** Sample characteristics for males (MG) and females (FG).

	MG	FG
Age (years)	24.7 ± 2.1	23.3 ± 0.5
BH (m)	1.80 ± 0.06	1.69 ± 0.07
BM (kg)	77.0 ± 10.48	62.0 ± 9.22
BMI (kg/m^2^)	23.72 ± 2.17	21.53 ± 1.81
SMM (kg)	37.92 ± 4.29	26.31 ± 4.31
PBF (%)	11.6 ± 4.14	22.45 ± 3.57
1RM BC: BM (a.u)	0.46 ± 0.10	0.27 ± 0.07
1RM PS: BM (a.u)	1.47 ± 0.18	1.27 ± 0.23

*BH* body height, *BM* body mass, *BMI* body mass index, *SMM* skeletal muscle mass, *PBF* percent of body fat, *1RM BC* BM—biceps curl one repetetition maximum: body mass ratio, *1RM PS* BM—parallel squat one repetetition maximum: body mass ratio.

Participants were healthy, without a history of upper or lower body musculoskeletal injuries. All participants were fully informed about the experimental procedures and potential risks and they signed a written informed consent prior to participation in the study. During the experimental period, the subjects were advised to stick to the usual diet and to avoid the use of supplementation. The study was approved by the Ethics Committee of the Faculty of Sport and Physical Education, University of Belgrade (ID number: 2316/19-2), performed in accordance with the Declaration of Helsinki and registered with ClinicalTrials.gov (NCT04845295, registration date: 14/04/2021).

### Elbow flexor and knee extensor strength assessment

Muscle strength was assessed by one-repetition maximum test (1RM) for two exercises: elbow flexion on Scott's bench (BC) and parallel barbell squat (PS). The testing was performed according to the protocol proposed by Baechle and Earle^33^: after a 10-min warm-up, the participants performed 8–10 repetitions with ~ 50% 1RM and 2–3 repetitions with 60–80% 1RM. Each participant had 5 attempts to lift the maximum weight with pauses between trials of 3 min.

BC testing was performed using a curling bar, where radio-ulnar joint was in the supination position. The axillae and back of the arms were positioned on the pad, while the height of the bench was adjusted for each participant until the trunk was straight and both feet were on the floor. Participants were required to accomplish a full range of movement^34^.

PS testing was performed with straight barbell bar which was placed above the acromion, with their feet shoulder-width apart. The range of motion of the exercise included a full concentric motion (until vertical position); during the eccentric phase the movement was performed until femur bones were parallel to the floor when the trochanter major and lateral epicondyle of femur were at the same level^35^.

### Elbow flexor and knee extensor dimensions assessment

Muscles dimensions were evaluated by an ultrasonic device (Siemens Antares, Erlangen, Germany), using the 2D ellipse diagnostic method, for 5 muscles: elbow flexor (biceps brachii muscle—BB) and 4 knee extensors (rectus femoris—RF; vastus intermedius—VI; vastus medialis—VM; vastus lateralis—VL).

Briefly, the measurements were performed while the subjects were seated with their elbows and knees extended and relaxed. The transducer, with variable high frequency (from 7 to 13 MHz), was held vertically with minimal pressure against the skin and water-soluble transmission gel was used between the transducer and the skin to ensure optimal image quality. The BB thickness was measured at two-thirds of the distance from the acromion to the antecubital crease. The muscle thickness (MT) included the distance in centimeters (cm) from the superficial to deep fascia layers and the average distance of the two measurements was used for statistical analysis^36^. The cross-sectional areas (CSA) of 4 knee extensor muscles were expressed in square centimeters (cm^2^). CSA of RF was measured at the height of proximal section of its distal third, above the musculoskeletal joint. CSA of VI and VM were measured at the height of distal part right above the patella^37^. The visible part of VL was measured at the distal third directly above the patella, under RF level. All the measurements were performed by the same specialized musculoskeletal radiologist.

### Elbow flexor and knee extensor contractile properties assessment

The contractile properties of BB, RF and VL muscles were evaluated by tensiomyography according to the manufacturer's instructions (TMG-BMC, Ljubljana, Slovenia). The values of contraction time (Tc) and radial displacement (Dm) were taken for analysis. The BB testing was performed while subjects were in a sitting position with the dominant arm bent at 90°. The tested arm was placed on a support, to ensure a neutral shoulder position during testing^25^. During the assessment of RF and VL contractile properties subjects were lying on the back. The dominant leg was placed on the support, forming a knee angle of 120°^23^.

Subjects were asked to perform a voluntary contraction, in order to mark the point of placement of the TMG sensor by the palpation method. Two self-adhesive electrodes (Pals Platinum, model 895220 with multi-stick gel, Axelgaard Manufacturing Co. Ltd) were placed proximal and distal at 3 cm from the marked point, emitting an electrical impulse. A sensor (GK40, Panoptik, Ljubljana, Slovenia) was placed between the electrodes to detect muscle changes initiated by electrical stimulation. The initial impulse was 25 mA and it increased proportionally by 10 mA, until the maximum (muscle no longer responds to electrical stimulus). The pause between the pulses was 10 s, to allow the muscle enough time to relax. The two best results were preserved and software calculated the mean^30^. Both TMG (pre-test–post-test) tests were conducted in the morning and by the same experienced specialist.

### Training intervention

Both experimental groups performed two exercises to target elbow flexor and knee extensor muscles: biceps curl on a Scott bench (Scott Bench-PA06, TechnoGym) and parallel barbell squat, respectively. All sessions were performed at the same time (13–15 h, room temperature 20°–24°), with a minimum 48 h rest between sessions on the same day each week. In both groups, the intensity of load (1RM%) and number of sets were as follows: in the first three weeks the subjects exercised with ~ 60% of 1RM in 3 sets, while during the next four weeks the load was set at ~ 70% of 1RM in 4 sets. All sets were performed until muscular failure. The pause between the sets was 2 min. Volume-load (repetitions × sets × weight lifted) has been recorded during the first and fourth week of training intervention for both MG (BC: 717.1 kg to 990.5 kg; PS: 2561.0 kg to 3339.0 kg) and FG (BC: 347.6 kg to 436.9 kg; PS: 1474.3 kg to 2442.5 kg).

### Statistical analysis

Test–retest repeatability for ultrasound, TMG and 1RM measurement were assessed using the intra-class correlation coefficient (ICC). Normality of data was tested by Shapiro–Wilk test, while homogeneity of variances and homogeneity of regression slopes were tested by Levene’s test and by interaction between the covariate and the independent variable, respectively.

Sex differences at baseline were tested using the independent t-test. One-way ANCOVA (using baseline values as covariates) was used to examine absolute differences in the tested variables, between the sexes. When ANCOVA showed statistical significance, the differences between groups were further estimated by Bonferroni post-hoc test. Relative changes for each variable were derived from *Pre* to *Post* percentage change for each participant. To determine sex differences in the relative changes, an ANCOVA model was applied, where a percentage change was used as a dependent variable and baseline values as the covariate. Effect sizes (ES) were determined using G-power software (University of Kiel, Kiel, Germany, version 3.1), based on the recommendations proposed by Rhea^32^ for untrained individuals; ES were considered as: trivial: < 0.50, small: 0.50–1.25, moderate: 1.25–1.90 and large: > 2.0.

Statistical analysis was processed using the IBM SPSS Statistics software package (Version 21, SPSS Inc, Chicago, IL, USA). All data presented by means ± SD. p ≤ 0.05 were taken as a statistically significant determinant.

## Results

Excellent reliability (all p < 0.01) was observed for ultrasound (BB: ICC = 0.997, CI = 0.986–0.999; RF: ICC = 0.997, CI = 0.990–0.999; VI: ICC = 0.995, CI = 0.979–0.998; VM = 0.997, CI = 0.987–0.999; VL: ICC = 0.996, CI = 0.982–0.999), 1RM (BS: ICC = 0.998, CI = 0.993–1.000; PS: ICC = 0.997, CI = 0.987–0.999) , TMG Tc ( BB: ICC = 0.928, CI = 0.713–0.982; RF: ICC = 0.955, CI = 0.825–0.989; VL: ICC = 0.939, CI = 0.765–0.985) and TMG Dm (BB: ICC = 0.951, CI = 0.804–0.988; RF: ICC = 0.951, CI = 0.815–0.988; VL: ICC = 0.969, CI = 0.884–0.992) measurements.

### Baseline measurement

At baseline measures, MG compared to FG demonstrated higher values of BB MT (p < 0.01; ES = 2.84) and CSA of RF (p < 0.05, ES = 1.20), VI (p < 0.01, ES = 1.97) and VM (p < 0.01, ES = 1.77). A significant difference was not found only for VL (p = 0.062, ES = 0.94), although in FG average CSA of VL was lower for about 0.55 cm^2^. Also, MG achieved significantly higher 1RM for BC (p < 0.01; ES = 3.20) and PS (p < 0.01; ES = 2.60). No sex-differences were observed for TMG parameters Tc and Dm (all p > 0.05).

### Training effects

**Figure 2:**
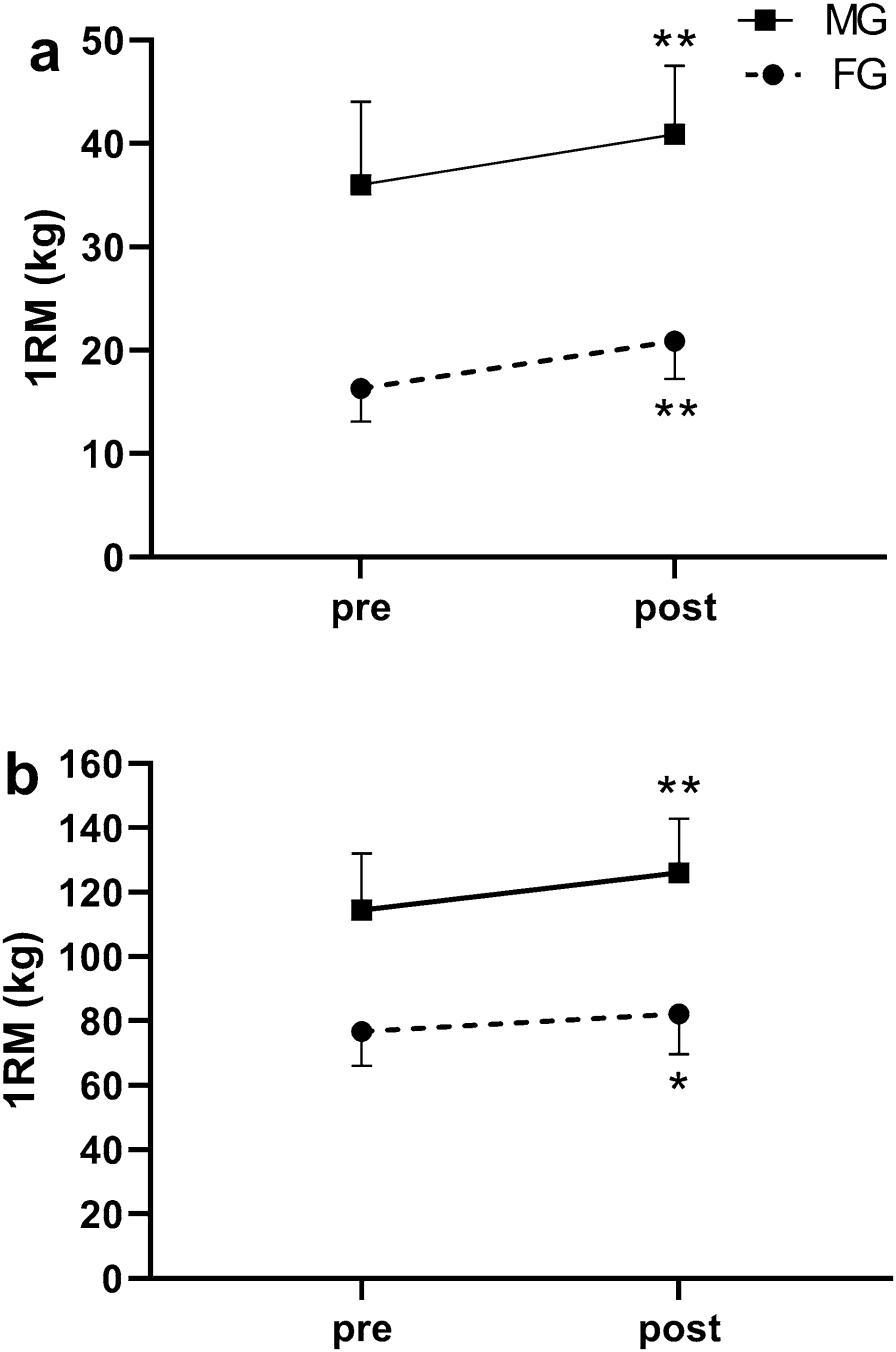
Pre-to-post changes in biceps curl (**a**) and parallel squat (**b**) one-repetition maximum (1RM) for males (MG) and females (FG). Graphical representations were generated using GraphPad Prism software version 9.0.2 (https://www.graphpad.com/scientific-software/prism/). *Significantly greater than pre-training (p < 0.05). **Significantly greater than pre-training (p < 0.01).

Both MG and FG significantly improved 1RM with a moderate-to-large effect for BC (MG for 4.9 ± 1.8 kg, p = 0.001, ES = 1.75 and FG for 4.5 ± 1.9 kg, p = 0.000, ES = 2.36) and PS (MG for 11.7 ± 6.6 kg, p = 0.001, ES = 1.77 and FG for 5.6 ± 5.2 kg, p = 0.013, ES = 1.08). The interactions sex × time was not significant neither for BC (F = 4.318, p = 0.055, ES = 0.53) nor PS (F = 3.108, p = 0.098, ES = 0.45) (Fig. 2). Although FG compared to MG displayed greater relative changes for BC and inversely MG compared to FG for PS, these differences were not significant when adjusted for pre-test values (Table 3).

Significant hypertrophy and small-to-moderate effect sizes were observed for BB (MG for 0.34 ± 0.21 cm, p = 0.001, ES = 1.62 and FG for 0.32 ± 0.16 cm, p = 0.000, ES = 2.00), RF (MG for 0.12 ± 0.07 cm^2^, p = 0.001, ES = 1.76 and FG for 0.22 ± 0.19 cm^2^, p = 0.010, ES = 1.14), VM (MG for 0.09 ± 0.07 cm^2^, p = 0.005, ES = 1.29 and FG for 0.14 ± 0.09 cm^2^, p = 0.001, ES = 1.65) and VL (MG for 0.14 ± 0.11 cm^2^, p = 0.005, ES = 1.26 and FG for 0.12 ± 0.08 cm^2^, p = 0.003, ES = 1.39). Only MG demonstrated a significant increase in CSA of VI muscle (MG for 0.20 ± 0.18 cm^2^, p = 0.008, ES = 1.16 and FG for 0.11 ± 0.16 cm^2^, p = 0.076, ES = 0.68). No significant sex × time interactions were observed for any tested muscle (Table 2). Analogously, there were no significant sex differences for the percent increase in any of the tested muscles (Table 3).

**Table 2:** ANCOVA output for sex-differences in absolute pre-to-post changes of muscle size.

	MG		FG		F	*p*	ES
	Pre	Post	Pre	Post			
BB MT (cm)	2.48 ± 0.45	2.82 ± 0.37**	1.51 ± 0.15	1.83 ± 0.21**	3.708	0.100	0.45
RF CSA (cm^2^)	3.84 ± 0.79	3.96 ± 0.76**	2.85 ± 0.83	3.08 ± 0.85**	0.851	0.371	0.24
VI CSA (cm^2^)	3.34 ± 0.83	3.54 ± 0.93**	2.04 ± 0.41	2.16 ± 0.50	0.366	0.554	0.16
VM CSA (cm^2^)	3.37 ± 0.68	3.46 ± 0.65**	2.42 ± 0.34	2.56 ± 0.38**	0.288	0.599	0.14
VL CSA (cm^2^)	3.75 ± 0.72	3.89 ± 0.69**	3.21 ± 0.35	3.33 ± 0.39**	0.491	0.594	0.18

*MG* males group, *FG* females group, *MT* thickness, *CSA* cross-section area, *BB* biceps brachii, *RF* rectus femoris, *VI* vastus intermedius, *VM* vastus medialis, *VL* vastus lateralis.

**Significantly greater than pre-training p < 0.01.

**Table 3:** ANCOVA output for relative sex-differences in tested variables using pre-test values as covariates.

	MG	FG	F	*p*	ES
	∆ (%)	∆ (%)			
BB CSA	14.7 ± 10.1	24.4 ± 10.8	0.971	0.340	0.25
RF CSA	3.5 ± 2.6	8.6 ± 8.7	0.377	0.549	0.16
VI CSA	6.0 ± 3.9	5.1 ± 7.2	0.846	0.372	0.24
VM CSA	3.0 ± 2.4	6.0 ± 3.5	0.473	0.502	0.17
VL CSA	4.0 ± 3.3	3.6 ± 2.5	0.574	0.461	0.20
1RM BC	15.1 ± 10.2	29.3 ± 15.9	0.699	0.416	0.22
1RM PS	10.7 ± 7.1	7.2 ± 6.7	2.804	0.115	0.43
BB Dm	− 12.2 ± 6.3	− 12.4 ± 5.4	0.001	0.996	0.01
RF Dm	− 18.5 ± 14.8	− 12.7 ± 15.9	0.631	0.440	0.21
VL Dm	− 8.2 ± 21.4	− 4.2 ± 16.9	0.193	0.667	0.12
BB Tc	2.6 ± 7.04	2.4 ± 14.5	0.244	0.628	0.12
RF Tc	5.6 ± 17.5	3.9 ± 14.9	0.011	0.918	0.03
VL Tc	2.6 ± 10.0	6.9 ± 16.9	1.942	0.185	0.37

*MG* males group, *FG* females group, *CSA* cross-section area, *1RM* one-repetition maximum, *Dm* radial displacement, *Tc* contraction time, *BB* biceps brachii, *RF* rectus femoris, *VI* vastus intermedius, *VM* vastus medialis, *VL* vastus lateralis.

**Figure 3:**
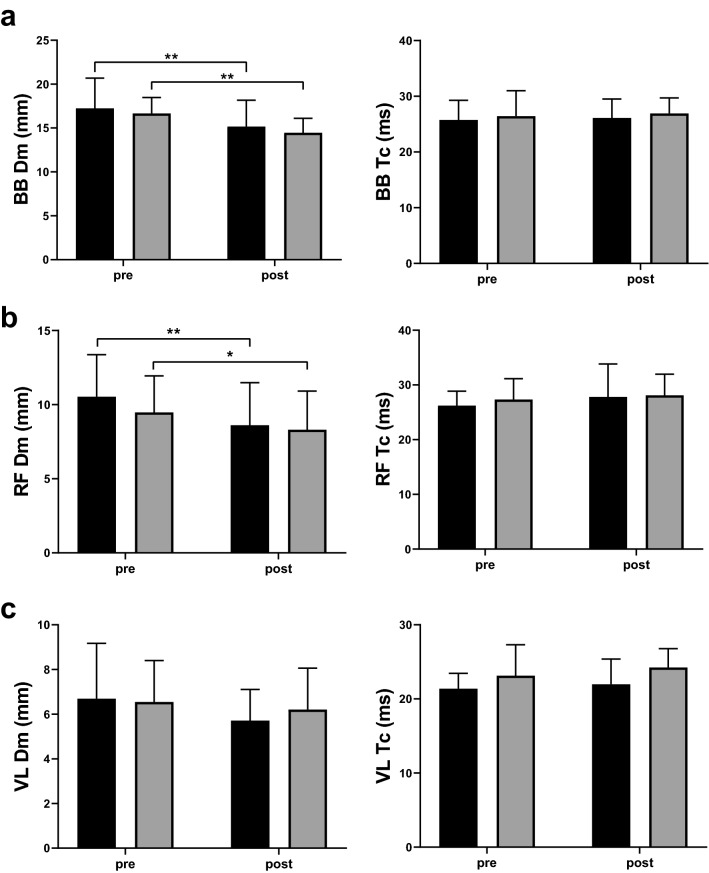
Pre-to-post changes in TMG parameters radial displacement—Dm (left) and contraction time—Tc (right) of biceps brachii (**a**), rectus femoris (**b**) and vastus lateralis (**c**) for males (black fill) and females (grey fill). Graphical representations were generated using GraphPad Prism software version 9.0.2 (https://www.graphpad.com/scientific-software/prism/). *Significantly greater than pre-training (p < 0.05). **Significantly greater than pre-training (p < 0.01).

TMG parameter Dm significantly decreased with a moderate-to-large effect for BB (MG for 2.1 ± 1.3 mm, p = 0.000, ES = 1.67 and FG for 2.2 ± 1.0 mm, p = 0.000, ES = 2.16) and a small effect for RF (MG for 1.9 ± 1.6 mm, p = 0.008, ES = 1.16 and FG for 1.2 ± 1.0 mm, p = 0.016, ES = 1.11) in both sexes, without significant sex x time interaction (BB: F = 0.246, p = 0.626, ES = 0.12, RF: F = 0.204, p = 0.658, ES = 0.12). Both sexes decreased Dm of VL muscle, however this did not meet statistical significance (MG for 1.0 ± 1.4 mm, p = 0.071, ES = 0.69 and FG for 0.4 ± 1.1, p = 0.367, ES = 0.34). Values of parameter Tc for all tested muscles remain unchanged in both sexes (all p > 0.05) (Fig. 3). Additionally, there were no significant differences between sexes for percent changes of TMG parameters (Table 3).

## Discussion

This study evaluated absolute and relative changes in size, strength and contractile properties of upper- and lower-body muscles with the general goal to give a comprehensive answer regarding sex-specific adaptations following RT. The main results indicate that 7 weeks of biceps curl and parallel squat exercises were a sufficient stimulus to promote hypertrophy and strength gains of biceps brachii and 4 knee extensor muscles, however there are no sex differences induced by RT intervention. Similar patterns were observed for TMG variables where both sexes equally increased muscle stiffness of biceps brachii and rectus femoris, while the values of contraction time of all tested muscles remain unaltered.

### Muscle strength

The present results demonstrate that, although males tend to display greater absolute strength gains, for relative changes there are no sex differences. While these results corroborate well the studies which investigated lower-body muscles^4,6–10^, they are in contrast with findings of a recent meta-analysis^15^, where females exceed males for relative upper-body strength changes. We believe that the reason for this discrepancy is two-fold and relates to the distinct experimental procedure and statistical approach.

Firstly, it should be noted that we applied a research design where 1RM testing was preceded by 2 weeks of familiarization protocol. Contrary, other studies conducted one trial session before strength testing^11–14^. Rapid increase in strength occurs during the initial 2–4 weeks of RT and it is primarily mediated by neural adaptations^4^. It has been hypothesized that females exhibit greater strength adaptations for upper-body due to pronounced skill acquisition and learning effects in the first weeks of RT, because untrained men are generally more familiar with the upper-body movements compared to the untrained women^15^. If this holds true, then there is a logical rationale that our 2-week familiarization period was sufficient to promote considerable neural adaptations and potentially compensate initial strength gains in females. This largely explains why we did not observe such dramatic differences between elbow flexor hypertrophy and strength changes in females (24% vs. 29%) as previous studies reported^12–14^.

Secondly, most of the results obtained from the aforementioned meta-analysis^15^, were based on comparing the means of percentage changes between sexes. For example, O'Hagan et al.^13^ reported greater relative strength increase in females based on the ANOVA analysis, while Dias et al.^11^ made these conclusions only by inspecting percentage differences between sexes (20% vs. 14%), without using any statistics. However, this approach could easily lead to the type I errors, considering that males and females differ on the baseline strength level and that the percentage changes will create bias towards the group with lower pre-test scores (in this case for females). On the other hand, a more suitable option is ANCOVA, where post-treatment scores are adjusted by the baseline values and which have the highest statistical power, especially when a group-baseline difference exists^38^. In that context, although we found similarly to O’Hagan et al.^13^ and Dias et al.^11^ a greater relative increase of biceps curl 1RM in FG compared to MG (29% vs. 15%), this was rather due to the lower baseline 1RM values in females than “true” differences in strength gains, as our ANCOVA analysis showed. This highlights that the interpretation of the results varies depending on the statistical methods used and we strongly believe that the ANCOVA model is the best fit option when aiming to compare sex adaptations following RT prescription.

### Muscle hypertrophy

One of the strengths of this study is that we included both upper- and lower-body muscles, and also evaluated dimensions of all 4 knee extensor muscles. Following RT, both sexes equally increased BB muscle size, which is consistent with previous reports^13–15^. From physiological standpoint, this is not surprising since the RT-induced protein synthesis and mTOR signaling pathway, which is considered as essential for muscle growth, do not differ between the sexes^39^. Although males generally demonstrate greater RT anabolic response^3^, current evidence suggests that circulating hormones are not associated with changes in muscle size^40^. On the other hand, given the existence of certain sex dissimilarities in lower-body anatomical level and muscle activity patterns^17–19^, we expected that squat RT would produce inhomogeneous hypertrophy of quadriceps muscles between MG and FG. However, this was not the case, as increase in CSA of quadriceps muscles was evident for both sex groups, without significant differences between them. As we mentioned earlier, there is a paucity of data regarding sex specific hypertrophy of individual quadriceps muscles and it is challenging to compare our results with previous reports. To the best of our knowledge, only Lundberg et al.^41^ explored inter- and intra-muscular adaptations of knee extensors produced by flywheel and convectional knee-extension RT. They also investigated sex-comparisons and found similar absolute changes in size of all quadriceps muscles between young men and women induced by both RT modalities. In the present study, we further demonstrate that squat RT produces similar absolute and relative increase in size of all quadriceps parts for both sexes and that there is no muscle-specific hypertrophy between males and females. Yet, hamstrings are a muscle group which could be of specific interest for future studies, given the recent findings of Mehls et al.^42^, who demonstrated greater activity of biceps femoris in men compared to women during squat exercise. Unfortunately, in the current study we did not evaluate other lower-body muscle groups except quadriceps femoris and future work is needed to clarify whether squat RT produces different hypertrophic response of hamstrings muscles between the sexes.

Interestingly, for both MG and FG, relative increase in size of BB muscle was greater than for all 4 quadriceps muscles (13–24% vs. 3–6%). Similar findings were obtained by both Cureton et al.^8^ and Abe et al.^4^, who reported greater hypertrophy of upper-body muscles compared to lower-body (10–20% vs. 4–8%). These discrepancies between upper and lower body hypertrophy could be related to different composition between muscles with parallel and pennate architecture. The BB muscle is largely composed of type II fibers and have higher numbers of parallel arranged sarcomeres^26^, hence they have a high capacity for producing force and higher mechanical tension, which in turn could lead to greater hypertrophy. On the other hand, relate to the slow twitch composition of the quadriceps muscles^26^, it is feasible that the increasing the training volume or time under tension could maximize resistance training hypertrophic effects on lower body muscle groups. Indeed, it has been shown that there is a dose–response relationship between weekly training volume and lower-body hypertrophy, and that it is necessary to perform a higher number of sets to maximize RT hypertrophic effects on lower muscle groups^43^. As we applied an identical RT prescription (number of sets) for both body regions (elbow flexor–knee extensor), there is a great possibility that a larger number of weekly sets (> 8) would lead to additional growth of quadriceps muscles.

Although not main goal of the study, of note is that the squat RT efficiently increased CSA of all quadriceps muscles. Conversely, it has been postulated that, given to the bi-articular role of RF, squat RT preferentially train vastii muscles among knee extensors^20^. However, findings on this topic are equivocal, where some authors^35,44^ did not observed significant hypertrophy of RF following squat training, while others^24,45^ found opposite results. Among other factors related to genetic predisposition and methods of muscle size assessments, this discrepancy in findings could be due to different PS technique. Specifically, our RT prescription included squats in high-bar position (at acromion level), while Kubo et al.^44^, applied a low-bar variant and did not found significant growth of RF. Unfortunately, other authors^24,35,45^ did not mentioned barbell placement during squats. Recently, it has been demonstrated that bar position highly affects activity of thigh muscles, where high-bar position provoke greater participation of RF during squat exercise, compare to the low-bar placement^46^. Hence, one could speculate that performing squat RT with a bar positioned at acromion level induce hypertrophy of all three vastii muscles and RF as well, and this should be investigated in further research.

### Tensiomyography

At baseline measurement we did not find significant sex differences in Dm for all tested muscles. This supports previous findings that muscle belly rigidness of quadriceps femoris is similar between the sexes in both sport^28^ and recreational^27^ population. Moreover, we further provide information that this is the case for elbow flexors (i.e. biceps brachii). Although no statistical significance was met, for both upper and lower-body muscles MG demonstrated lower Tc values. As Tc could be related to muscle fiber type composition^26^, there is a good possibility that lower values of Tc represent a higher percentage of fast twitch-fibers in males. A well-designed cross sectional research, with a larger sample size and biopsy evaluation, could give a more precise answer regarding this topic.

To the best of the authors’ knowledge, this is the first study to compare TMG longitudinal adaptations between males and females. Comparable to hypertrophy and strength gains, both sexes demonstrated similar changes in TMG parameters after 7 weeks of the training protocol. It has been shown that RT leads to reduced Dm values, in both acute and chronic cases^23,24,30^. Consistent with these reports, we found a chronic decrease in Dm values for both MG and FG, which implies that RT leads to an increase in muscle stiffness equally for both sexes. Interestingly, we found that RT reduced Dm for both BB and RF muscles, while for VL there were no significant changes. This is also observable in the study of Wilson et al.^24^, who reported a significant decrease in the Dm value for RF and not for VL muscle, after 8 weeks of squat RT. Furthermore, neither plyometric training elicited significant stiffness of VL, in contrast to biceps femoris muscle^30^. These findings could be explained by the fact that VL is a postural and already highly stiffed muscle, which was shown by baseline measurement (where Dm values of VL were lower compared to BB and RF), and the training stimulus does not cause such drastic changes as it is the case with non-postural muscles. In addition, parameter Tc remains unaltered following training intervention for all tested muscles, which indicates that there are no chronic changes in muscle contraction time induced by RT.

### Limitations

Finally, we are aware that our research may have several limitations. Firstly, we measured muscle dimensions at one site only. Since muscle growth can be non-uniform along the length^35^, we cannot rule out the possibility that greater hypertrophy occurred at other regions of muscles. Secondly, training intervention lasted only 7 weeks. Although this period is sufficient to produce significant muscular changes in untrained subjects^47^, it is not clear whether males and females would demonstrate different adaptations for a longer duration of time. Thirdly, the menstrual cycle of female participants has not been monitored. Yet, based on the recent findings^48^ ovulation period does not appear to influence muscle size and strength in women. Fourthly, although we advised study participants to stick to their usual diet, we did not systematically follow up their nutrition. Lastly, we applied a different ultrasound estimation techniques (MT/CSA) for biceps brachii and quadriceps muscles.

## Conclusion

Taken together, we can conclude that there are no specific sex differences in morphological, functional and contractile muscle adaptations produced by RT either for upper- nor lower-body muscles. Therefore, exercise prescriptions should not be prioritized on sex characteristics. From the hypertrophy standpoint, parallel barbell squat is a highly effective exercise to promote significant growth of all quadriceps muscles. Additionally, TMG parameter Dm is chronically altered by the RT program and the increase in muscle stiffness is similar for both sexes.
